# Classification framework to identify similar visual scan paths using multiple similarity metrics

**DOI:** 10.16910/jemr.17.3.4

**Published:** 2024-08-09

**Authors:** Ricardo Palma Fraga, Ziho Kang, Jerry M. Crutchfield

**Affiliations:** University of Oklahoma, United States; Federal Aviation Administration, United States

**Keywords:** eye movement, scan path, gaze, eye tracking, air traffic control, tower control, string edit algorithm, Jaccard coefficient similarity

## Abstract

Analyzing visual scan paths, the time-ordered sequence of eye fixations and saccades, can help us understand how operators visually search the environment before making a decision. To analyze and compare visual scan paths, prior studies have used metrics such as string edit similarity, which considers the order used to inspect areas of interest (AOIs), as well as metrics that consider the AOIs shared between visual scan paths. However, to identify similar visual scan paths, particularly in tasks and environments in which operators may apply variations of a common underlying visual scanning behavior, using solely one similarity metric might not be sufficient. In this study, we introduce a classification framework using a combination of the string edit algorithm and the Jaccard coefficient similarity. We applied our framework to the visual scan paths of nine tower controllers in a highfidelity simulator when a “clear-to-take-off” clearance was issued. The classification framework was able to provide richer and more meaningful classifications of the visual scan paths compared to the results when using either the string edit algorithm or Jaccard coefficient similarity.

## Introduction

Investigating how operators visually search the environment, such as what information they observe (or don’t observe), as well as the order they observe said information, can help us better understand their decision-making process (20; 34; 21; 23; 37; 26). A common way to study how visual search takes place is by creating and analyzing visual scan paths, the time-ordered sequence of eye fixations and saccades (16) collected by an eye tracking device. In the field of air traffic control, visual scan paths have been analyzed to explore the visual search patterns and conflict-mitigation strategies of en-route controllers (17; 27; 24; 32) as well as local tower controllers (4; 18).

Two approaches to analyze visual scan paths are to evaluate them based on the order information was observed or based on what information was observed (33). To evaluate based on the order of information was observed, such as the order of areas of interest (AOIs) in the environment were inspected, prior studies have used measures such as string edit similarity. The string edit similarity calculates the number of transformations (i.e., insertions, deletions, and substitutions) needed to convert one visual scan path sequence into another visual scan path sequence (33; 9). On the other hand, to evaluate visual scan paths based on what information was observed without considering the order the information was observed in, prior studies have compared the AOIs in common between two visual scan paths (33; 7). One way researchers have carried out these comparisons has been by applying measures such as the Jaccard coefficient similarity (19; 3). Note that both the string edit similarity and the Jaccard coefficient similarity are explained in more detail in the *Methods* section.

**Figure 1:**
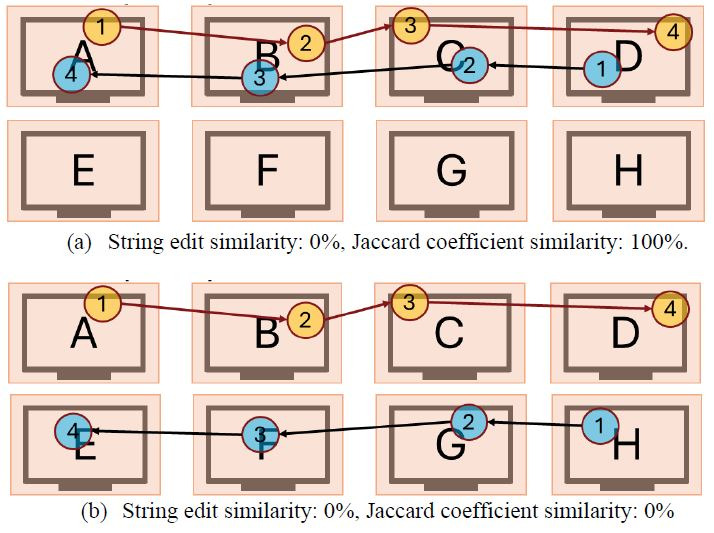
Representative example showcasing how using multiple visual scan path similarity metrics might provide additional information to help identify similar visual scan paths. Note: Figure 1(a) showcases two visual scan paths that observed the same AOIs in the oppo-site order, resulting in a 100% Jaccard coefficient similarity but a 0% string edit similarity. On the other hand, Figure 1(b) includes two visual scan paths that observed completely different AOIs, resulting in both a 0% Jaccard coefficient similarity and a 0% string edit similarity.

However, using solely one similarity metric might not be sufficient to identify similar visual scan paths. Consider the two sets of visual scan paths showcased in Figure 1 below. One can observe how the string edit similarity between the visual scan path sequences in Figure 1(a) (ABCD and DCBA) and Figure 1(b) (ABCD and HFGE) are both 0%, leading us to the interpretation that the two visual scan paths did not follow a similar order in either case. However, including the Jaccard coefficient similarity highlights a key difference between the visual scan paths in both examples. The visual scan paths in Figure 1(a) inspected the same AOIs (A, B, C, and D, resulting in 100% Jaccard coefficient similarity) but applied a different order when doing so, while the visual scan paths in Figure 1(b) inspected different AOIs (resulting in 0% Jaccard coefficient similarity). One visual scan path inspected the AOIS A, B, C, and D while the other inspected the AOIs E, F, G, and H. As a result, using multiple similarity metrics might provide additional information that could help provide a more complete picture as to how two visual scan paths are similar to each other than when only one similarity metric is used.

One task in which information might be gathered in a different order or from different sources that provide the same or similar information is tower air traffic control. A prior study discussed how a tower controller issuing “continue approach” clearances observed the same information each time but in a different order (36). In a separate study, researchers highlighted that tower controllers might “add[ing] additional elements or repeat[ing] patterns differently” throughout their visual scan paths (28). As a result, using only one similarity metric to compare visual scan paths might not fully capture the possible visual scan path variations between tower controllers (i.e., adding or removing elements, repeating patterns differently) that apply a similar visual scanning behavior. Thus, making it challenging to identify tower controllers that apply a similar visual scanning behavior from those that use a different one.

As a result, the present study introduces a visual scan path sequence comparison framework to classify similar visual scanning behaviors by combining two similarity measures: one that consider the order information was gathered (i.e., the order of AOIs in the visual scan path), and another that considers what information was observed (i.e., the AOIs in the visual scan path). More specifically, the string edit similarity and Jaccard coefficient similarity between visual scan paths. Our approach calculates the average similarity values for each metric between all visual scan paths and uses those values as thresholds to classify visual scan paths as either highly similar across both metrics, highly similar in one metric, or not similar in either metric. The proposed approach was applied to identify similar visual scan paths carried out by expert tower controllers as they issue clear to take off clearance in a high-fidelity tower cab simulator.

## Proposed framework

The proposed framework is explained in the following three sections. First, we describe the two similarity metrics used to compare visual scan paths based on the order of AOIs, as well as based on the AOIs inspected. Second, we introduce the proposed classification procedure to classify visual scan paths based upon their similarity metric values. Lastly, we describe how to identify similar visual scan paths based on their average similarity values. Each section contains worked out examples to showcase the steps of the proposed procedure.

### A. Calculating similarity between visual scan paths

To calculate the similarity between visual scan path based on the AOIs present in both scan paths we apply the Jaccard similarity coefficient (14; 11). The Jaccard similarity coefficient has previously been used in multiple different domains, such as clustering (35), computational biology (1), as well as eye tracking (19; 3). The Jaccard similarity coefficient defines similarity by calculating the number of elements in common between two sets divided by the number of elements in total present in both sets. In other words, in the context of visual scan paths, the number of AOIs common between two scan paths (i.e., the intersection of AOIs) divided by the total number of AOIs present in both scan path sequences (i.e., the union of AOIs) as can be observed in equation (1).

(1) $$J\left(A,B\right)=\frac{\left|A\cap B\right|}{\left|A\cup B\right|}$$

Here, A represents the set of AOIs present in one scan path sequence, and B the set of AOIs present in another scan path sequence, A∩B contains the number of AOIs in common between the two scan path sequences, and A∪B includes the number of AOIs present in both scan path sequences. Consider the scan path sequence A = *EGHJ* and the scan path sequence B = *JEOG.* Here, the value of A∩B would be 3, as there are 3 AOIs in common between the two scan path sequences (i.e., EGJ), while A∪B would be 5, as there are 5 AOIs in total present between the two scan path sequences (i.e., H and O in addition to EGJ). Therefore, the Jaccard coefficient similarity between these two scan path sequences would be $J\left(A,B\right)=\;\frac{3}{5}=0.6.$

On the other hand, to calculate the similarity between visual scan paths by considering the order of AOIs, we apply the string edit similarity metric (22). The string edit similarity metric has commonly been used in multiple eye tracking studies to calculate the similarity between scan paths (33; 7; 5). As mentioned previously, the string edit algorithm computes the number of operations (insertions, deletions, and substitutions), known as the string-edit distance, required to transform one scan path sequence into another. We defined the normalized string edit similarity as done by Privitera & Stark (33), where the string edit distance is converted into similarity by subtracting one from the string edit distance value calculated, and normalized by dividing by the length of the longest scan path sequence, as can be observed in equation (2) below.

(2) $$S\left(A,B\right)=1-\left(\frac{i+d+s}{n}\right)$$

In this equation, A represents one scan path sequence while B another scan path sequence. The variables i, d, and s indicate the number of insertions, deletions, and substitutions, respectively, needed to convert the scan path sequence A into B. Lastly, n represents the length of the largest scan path sequence between A and B. Consider as an example the scan path sequence A = *EFD* and the scan path sequence B = *EFCD.* To convert the scan path sequence A into the sequence B, one must insert the AOI C into scan path sequence A. Thus, only one insertion operation is needed. Given the length of the largest scan path sequence is 4, the normalized string edit similarity would be $S\left(A,B\right)=1-\left(\frac{1}{4}\right)=0.75$.

In addition, note that the string edit similarity and Jaccard coefficient similarity metrics share an inherent relationship to each other. For the order of AOIs to be considered (using the string edit similarity metric), said AOIs must first be inspected (using the Jaccard coefficient similarity) by the participant. As a result, large (or low) Jaccard coefficient similarities values might also result in large (or low) string edit similarity values. However, given that string edit similarity is primarily influenced by the order and location of AOIs in the sequence, while the Jaccard coefficient similarity does not consider the order nor location of AOIs. Thus, two visual scan paths might have a large Jaccard coefficient similarity but a low string edit similarity or vice versa.

### B. Classifying visual scan paths based on similarity metrics

**Figure 2:**
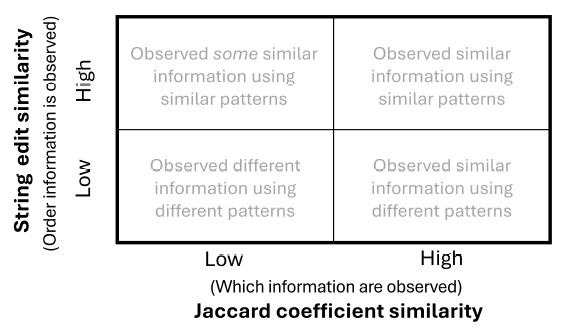
Classification of scan path sequences based on Jaccard coefficient similarity and string edit similarity values.

The proposed framework compares visual scan paths by calculating their Jaccard coefficient similarity and string edit similarity and classifies them into four categories (Figure 2): (1) Observed similar information using similar patterns (high string edit similarity and high Jaccard coefficient similarity); (2) Observed *some* similar information using similar patterns (high string edit similarity and low Jaccard coefficient similarity); (3) Observed similar information using different patterns (low string edit similarity and high Jaccard coefficient similarity); (4) Observed different information using different patterns (low string edit similarity and low Jaccard coefficient similarity).

Visual scan path sequences that contain high values on both similarity metrics are considered to represent similar visual scanning strategies, while those that possess low similarity across both similarity metrics are considered to be different visual scanning strategies. When the two similarity metrics differ, we consider the two visual scan paths to be variations of a similar underlying visual scanning behavior. More specifically, in the case of a high Jaccard coefficient similarity but a low string edit similarity, the visual scan paths contained a high number of AOIs in common but had different patterns when inspecting the AOIs. On the other hand, when the Jaccard coefficient similarity is low and the string edit similarity is high, the visual scan path contained fewer common AOIs but used more common patterns when inspecting the AOIs in common.

**Figure 3:**
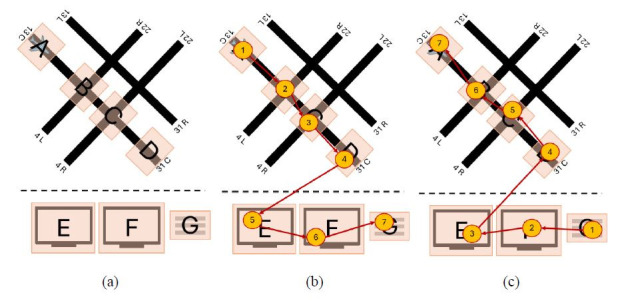
Simplified example of impact two visual scan paths (b) and (c) classified as having high string edit similarity but low Jaccard coefficient similarity on an airport environment (a). Note: The airport drawn by the researcher resembles the Midway International airport. AOIs were drawn on potential hotspots, locations outside the tower cab where aircraft most frequently cross and where errors might have severe consequences (4), defined by the researchers. These hotspot AOIs included the beginning and end of the runway (AOIs A and D), as well as intersecting runways (AOIs B and D). In addition, AOIs E and F were drawn to represent radar screens present in the tower environment, while AOI G represents the flight strips, which contain information (e.g., destination airport) regarding the aircraft. Furthermore, Figure 1(b) highlights the visual scan path of one controller, creating the scan path sequence ABCDEFG; Figure 1(c) showcases a similar visual scan path of another controller but in the opposite order, creating the scan path sequence GFEDCBA. The yellow circles denote AOIs inspected, and the inscribed numbers the order the AOIs were inspected in. The red lines represent the order in which the AOIs were inspected.

**Figure 4:**
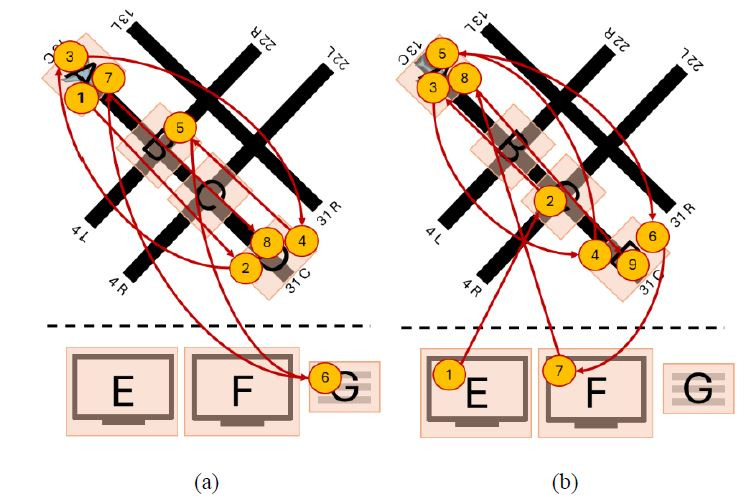
Simplified example of two visual scan paths (a) and (b) classified as having high string edit similarity and low Jaccard coefficient similarity. Note: The yellow circles denote AOIs inspected, and the inscribed numbers the order the AOIs were inspected in. The red lines represent the order in which the AOIs were inspected.

**Figure 5:**
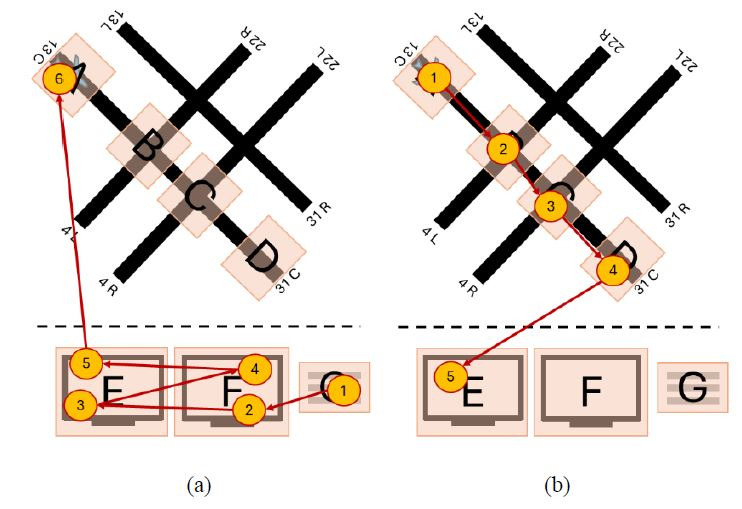
Simplified example of two visual scan paths (a) and (b) classified as having low string edit similarity and low Jaccard coefficient similarity. Note: The yellow circles denote AOIs inspected, and the inscribed numbers the order the AOIs were inspected in. The red lines represent the order in which the AOIs were inspected.

**Figure 6:**
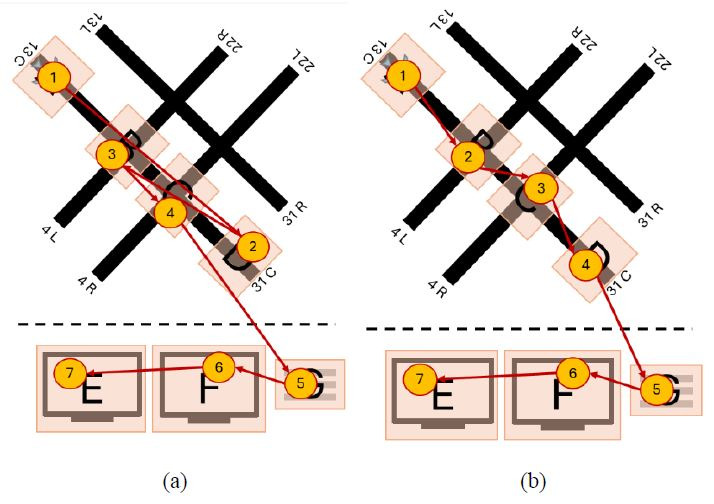
Simplified example of two visual scan paths (a) and (b) classified as having high string edit similarity and high Jaccard coefficient similarity. Note: The yellow circles denote AOIs inspected, and the inscribed numbers the order the AOIs were inspected in. The red lines represent the order in which the AOIs were inspected.

To visualize the application of the proposed classification framework, consider the following examples in Figures 3, 4, 5, and 6 explained in more detail below.

Figure 3 showcases how two controllers inspected all of the same AOIs but in reverse order. More specifically, Figure 3(b) contains the sequence ABCDEFG, while Figure 3(c) showcases the sequence GFEDCBA. As a result, the Jaccard coefficient similarity between these two scan path sequences is high (1), as they inspected all the same AOIs in the airport environment, but the string edit similarity is low (0.143), due to the different (i.e. reverse) order.

Figure 4 contains two visual scan paths where controllers inspected different AOIs (B and G in Figure 4(a) and C, E, and F in Figure 4(b)) but had similar patterns among the few AOIs they had in common (multiple movements between AOI A and D), resulting in a high string edit similarity (0.556) but low Jaccard coefficient similarity (0.286).

Figure 5 includes two controllers applying different visual scanning strategies, where one controller (Figure 5(a)) focused primarily on the radars (AOIs E and F) and flight strip (AOI G), while the other controller scanned the runway hotspots (AOIs A-D) and only one of the radar screens (AOI E). As a result, these two visual scanning strategies have low string edit similarity (0.167) and low Jaccard coefficient similarity (0.286).

Figure 6 includes two controllers applying similar visual scanning strategies. Both controllers follow a nearly identical order when scanning the active runway hotspots (AOIs A-D), followed by scanning the radars (AOIs E and F) and the flight strips (G). As a result, these two visual scanning strategies have high string edit similarity (0.714) and high Jaccard coefficient similarity (1).

### C. Identifying similar visual scan paths among multiple individuals

To identify similar visual scan paths, our proposed framework calculates the average Jaccard coefficient similarity and average string edit similarity values between all visual scan paths, which are then used as thresholds to classify visual scan paths as having “high” and “low” similarity. Using such an approach allows us to classify visual scan paths without making assumptions about how similar (or dissimilar) visual scan paths are for any one task. Previous research has discussed how the task to be completed might affect how similar the visual scan paths of participants are to each other (7). For example, in a task where participants are instructed to look in the environment in a specific order (e.g. following the movement of a dot), the string edit similarity between visual scan paths might be higher than in a task where participants are able to freely search the environment. Thus, “high” and “low” similarity visual scan paths can be identified based on the visual scanning behavior contained in each controller’s visual scan path, without requiring the researcher to define “high” and “low” similarity thresholds based on their subjective judgement.

**Figure 7:**
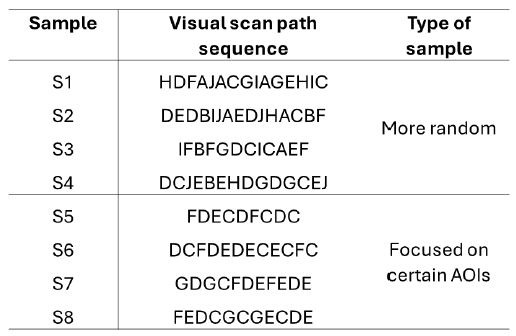
Visual scan path sequences created by sampling 10 AOIs (A, B, C, D, E, F, G, H, I, and J) with either uniform probabilities (type of sample: More random) or by assigning specific probabilities to each AOI (type of sample: Focused on certain AOIs)

To showcase an application of the proposed framework, 8 simulated visual scan paths (Figure 7) were created by sampling 10 AOIs (A, B, C, D, E, F, G, H. I, and J) a total of 15 times each. Four of the simulated visual scan paths had an equal probability of observing any AOI (i.e. all 10 AOIs had an equal 10% probability of being selected), and thus, showcased a more random visual search pattern. On the other hand, the remaining four simulated visual scan paths had specific sampling probabilities assigned to each AOI (A: 0%, B: 0%, C: 20%, D: 20%, E: 30%, F: 25%, G: 5%, H: 0%, I: 0%, J: 0%), and thus, the visual scan paths focused on certain AOIs (C, D, E, and F) more than others. Note that if the same AOI was sampled consecutively, only one sample was kept (i.e. AAABC would be converted as ABC), and thus, the length of some visual scan paths may be less than 15 AOIs.

The proposed framework classified the four visual scan paths that focused on specific AOIs as highly similar across both similarity metrics, and classified the remaining four random visual scan paths as having low similarity across both similarity metrics (Figure 6(a-b)). Thus, distinguishing between the visual scan paths that showcased a particular strategy of focusing on certain AOIs (which contained multiple transitions between AOIs D and E, E and C, as well as AOIs G and C) from those that treated all AOIs as equally important and were thus more random.

**Figure 8:**
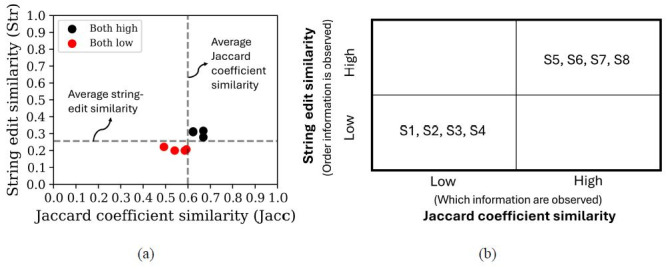
Results of the application of the proposed framework to the simulated visual scan paths Note: Figure 8(a) visualizes the string edit and Jaccard coefficient similarities of each simulated visual scan path sample, as well as their classification color (where black dots represents visual scan path sequences classified as high across both metrics, red dots denote visual scan path sequences classified as low across both metrics). Figure 8(b) showcases the classification results as depicted in the proposed classification framework.

## Methods

### A. Participants and Apparatus

A total of 14 retired local controllers, controllers in the tower cab responsible for aircraft entering, leaving, and crossing runways (8), with an average of 26 years of experience (range between 10 and 42 years) participated in the experiment. However, data from 5 participants was not included in the present work due to low eye tracking data quality, as some eye movements carried out by the participants were not collected by the eye tracker. As a result, only data from 9 participants was analyzed in the present work.

The controllers managed simulated landing and departing air traffic on a high-fidelity Adacel tower simulator used at the FAA’s Civil Aeronautical Medical Institute (CAMI) in Oklahoma City, Oklahoma. Twelve 55” HD (1080p) monitors, wrapped greater than 180*°* around the participant, were used to simulate the out the window view of tower cab. The simulators included flight strips of the aircraft in the scenarios, as well as working Bright Radar Indicator Terminal Equipment (BRITE) and Airport Surface Detection (ASDE) radar displays. The participants issued verbal clearances to the aircraft using a standard communication headset.

The Tobii Pro Glasses II (100 Hz) were used to capture participant eye movements (equipped with prescription lenses as necessary). In addition, the Tobii Pro Lab software was used to visually observe the recorded video from the participant’s point of view that contained the raw gaze data overlaid in order to verify the accuracy of the gaze points, as well as to apply the I-VT algorithm in order to identify eye fixations and saccadic movements, which were used to create the visual scan path sequences. These processes are explained in more detail in the processing eye movement data section below.

### B. Task and Scenario

The participants were tasked with managing landing and departing air traffic during a scenario that lasted approximately 22 minutes. The scenario took place during daylight hours with unlimited visibility and clear skies. Furthermore, the scenario contained a total of 33 aircraft, with 19 arriving aircraft and 14 departing aircraft. The scenario ended once the 22-minute mark was reached, which might occur prior to all aircraft present in the environment being issued a clearance.

**Figure 9:**
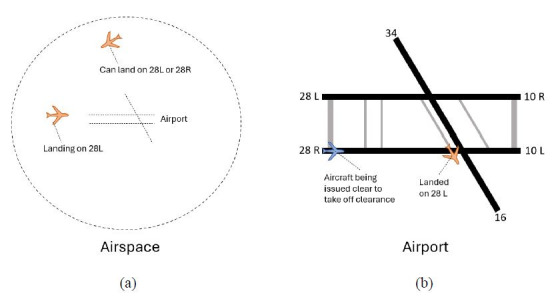
Visualization of the operational environment of the airspace and airport while the clear to take off clearance analyzed was issued. Note: The aircraft that will be issued the clear to take off clearance is highlighted in blue, while a total of three aircraft of interest to the participant are highlighted in orange. Two of the aircraft of interest in the airspace are arriving at the airport in the next few minutes, with one aircraft already issued a clear to land clearance to runway 28L. The other aircraft of interest has already landed and is exiting the runway momentarily.

The present study focuses on the first takeoff clearance issued by all the participants to account for potential factors that might impact the similarity between controllers (e.g., location of aircraft on the airport). The location of aircraft in the environment can be observed in Figure 9. In the airspace (Figure 9(a)), there were two aircraft present (orange aircraft), one that had already been issued a landing clearance by the controller, and a second aircraft that was approaching the airport. On the ground (Figure 9(b)), there were two aircraft present, one that recently landed and was currently (or close to) exiting runway 28R (orange aircraft), while another aircraft (blue aircraft) was waiting to be issued the clear to take off clearance by the controller. Note that other aircraft that were present in the airport environment, such as those taxiing towards a runway for departure, are under the control of the ground controller.

### C. Processing eye movement data

The eye movements of participants were analyzed while issuing a single a clear to take off clearance to one aircraft, where controllers are more likely to show focused attention (25). In this study, the time to issue the clearance began from the moment the aircraft reports to the tower controller that it is ready for departure up until the point when the controller finishes issuing the clearance. Among all 9 participants, the average time to issue the clear to take off was approximately 25.1 seconds, with the lowest time being 15.7 seconds while the longest was 32.2 seconds.

The I-VT algorithm implemented in Tobii Pro Lab was used to identify eye fixations and saccadic movements. The gaze velocity threshold of the I-VT algorithm was set at 90 °/s and all other settings were left at their default values (e.g., minimum eye fixation duration of 60 ms) (31). The 90 °/s threshold value was selected and validated by the researchers by ensuring that the eye movements of participants were accurately represented by observing the video recorded by the Tobii Pro Glasses II with the participants’ gaze movements overlaid. Furthermore, the collected gaze samples were manually checked by observing the video recorded by the Tobii Pro Glasses II overlaid with the gaze samples. An automated data processing carried out by the Tobii Pro Lab software is to map gaze samples to a snapshot containing the AOIs in order to identify whether the gaze took place within the AOI (38). During the process, gaze samples might be erroneously placed at an incorrect AOI, when they actually took place on another AOI, or might not be mapped at all in the first place. The process requires checking each gaze collected by the eye tracking device, requiring a manual inspection of hundreds of gaze points even for very short periods of data collection, making this process a time consuming task (29).

**Figure 10:**
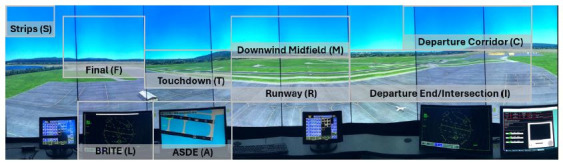
The high-fidelity tower control simulator used in the experiment with the areas-of-interest (AOIs) highlighted (gray squares). Note: All AOIs were represented as individual letters in the scan path sequences of participants in order to calculate Jaccard coefficient and string edit similarity values. The letters assigned to each AOI are indicated in the figure (e.g.., Final = F, Strips = S).

AOIs in the tower cab environment (Figure 10) were identified and created in collaboration with subject matter experts based on their operational significance. The AOIs included the active runways (Runway AOI), as well as the two hotspots in the active runway (Touchdown AOI and Departure/End Intersection AOI), the radars contained within the tower cab (ASDE AOI and BRITE AOI), arrival and departure corridors (Final AOI and Departure Corridor AOI), as well as the downwind area that arrival aircraft must cross prior to their final approach (Downwind Midfield AOI). Finally, gazes on the flight strips, which contain information regarding the aircraft (e.g., callsign, destination airport), as well as on notes written by participants on a notepad (e.g., sequence of aircraft departing) were manually mapped using the Tobii Pro Lab software to the upper left corner (Strips AOI) of the image.

Lastly, AOIs were included in the participant’s visual scan path sequence if at least one eye fixation took place within the AOI. Thus, if a controller inspected the Strips AOI followed by the BRITE AOI and the Runway AOI the visual scan path sequence would be Strips -> BRITE -> Runway. Note that each AOI was assigned a unique letter to represent them in the visual scan path sequence, which can be observed in Figure 10 (e.g. the Departure Corridor is represented by the letter C). As a result, the visual scan path sequence Strips AOI (S) -> BRITE AOI (L) -> Runway AOI (R) would be represented as SLR.

### D. Data analysis

The proposed framework was applied by calculating the string edit similarity and Jaccard coefficient similarity between the participants’ visual scan paths sequences. The average Jaccard coefficient and string edit similarity values between all visual scan path sequence comparisons were used to define “high” and “low” similarity values in order to classify visual scan path sequences using the matrix shown in Figure 1. Lastly, the visual scan paths of participants were visualized by groups based on their assigned classification and compared to identify visual scanning behaviors.

## Results

**Figure 11:**
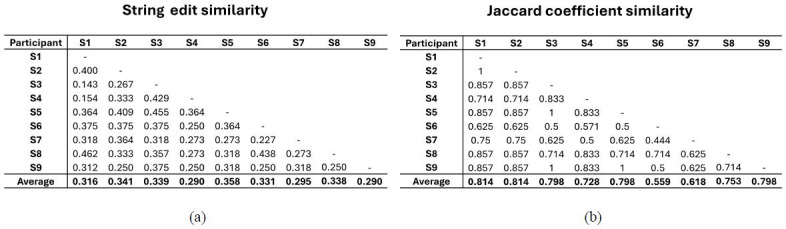
Individual and average (a) string edit similarity values and (b) Jaccard coefficient similarity values calculated across visual scan path comparisons.

Figure 11 contains the string edit similarity and Jaccard coefficient similarity values calculated between the scan path sequences of the tower controllers. On average, the Jaccard coefficient similarity values between participants (0.742) were higher than the average string edit similarity values (0.322). The highest Jaccard coefficient similarity value achieved between two visual scan paths was 1 while the lowest value was 0.444. For the string edit similarity, the highest value calculated was 0.462 and the lowest value was 0.143.

**Figure 12:**
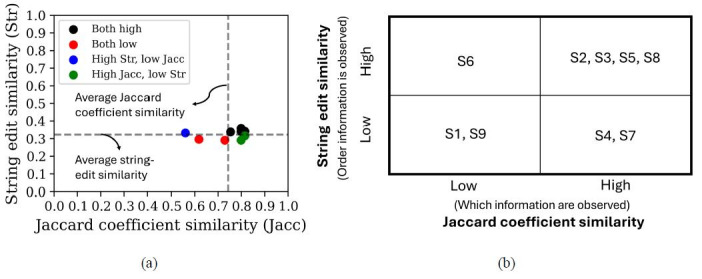
Classification matrix of the 9 visual scan paths of expert tower controllers while issuing a clear to take off clearance based on their average string edit and Jaccard coefficient similarity values. Note: Figure 12(a) visualizes the string edit and Jaccard coefficient similarities of each visual scan path as a scatterplot (where black dots represents visual scan path sequences classified as high across both metrics, red dots denote visual scan path sequences classified as low across both metrics, blue dots contain those visual scan paths classified as high string edit similarity but low Jaccard coefficient similarity, and lastly, red dots depict those visual scan path sequences with high Jaccard coefficient similarity but low string edit similarity). Figure 12(b) showcases the classification results as depicted in the proposed classification framework.

Figure 12(a-b) showcases the classification of visual scan paths based on the average string edit and Jaccard coefficient similarity values between participants. Most visual scan paths of controllers were classified as highly similar in at least one similarity metric. More specifically, four visual scan paths were classified as highly similar across both similarity metrics, one visual scan path was classified as having high string edit similarity but low Jaccard coefficient similarity, and the remaining two visual scan paths were classified as having low string edit similarity but high Jaccard coefficient similarity. The remaining two visual scan paths were classified as having low similarity across both similarity metrics.

**Figure 13:**
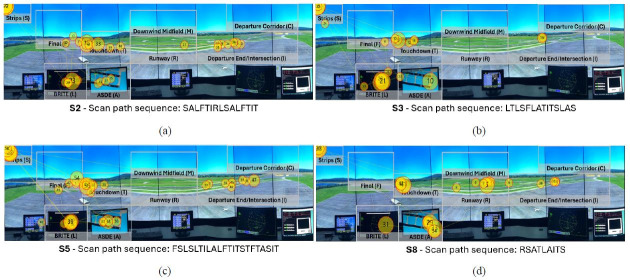
Visualization of four visual scan paths (a-d) classified as highly similar across both string edit and Jaccard coefficient similarities overlaid the AOIs in the airport environment, alongside their scan path sequences. Note: The yellow circles denote eye fixations, and the inscribed numbers the order the said eye fixations took place. The yellow lines represent the saccadic movement of participants between eye fixations.

The visual scan paths classified as highly similar across both similarity metrics can be observed in Figure 13. All visual scan paths inspected the two runway hotspots AOIs (T and I), as well as the flight strips AOI (S) and both the BRITE radar (L) and the ASDE radar (A). Two of the four visual scan paths contained the runway AOI (R), while three of the four visual scan paths contained the Final (F) AOI. Furthermore, the scan path sequences showcase common patterns between the controllers. More specifically, two visual scan paths (S5 and S3) had the same pattern TITS to inspect the runways and the flight strips, while the remaining two controllers had a very similar variations, such as TIT (S2) and ITS (S8). Similarly, all visual scan paths contain common patterns between the radar AOIs, such as LA or AL, as well as with the flight strips AOI, such as AS or SA.

**Figure 14:**
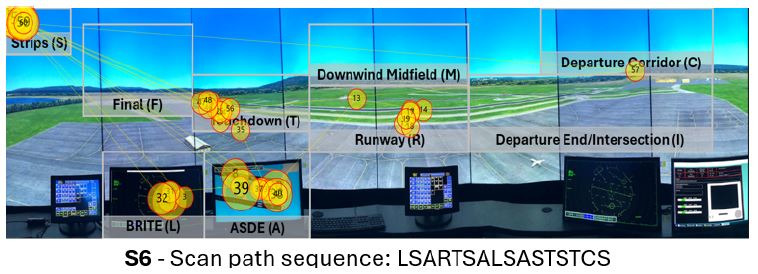
Visualization of the visual scan path classified as having high string edit similarity but low Jaccard coefficient similarities overlaid the AOIs in the airport environment, alongside their scan path sequence. Note: The yellow circles denote eye fixations, and the inscribed numbers the order the said eye fixations took place. The yellow lines represent the saccadic movement of participants between eye fixations.

Figure 14 visualizes the sole visual scan path classified as having high string- dit similarity but low Jaccard coefficient similarity. The visual scan path did not contain two commonly inspected AOIs, the Final AOI (F) as well as the Intersection AOI (I). Furthermore, it is the only visual scan path that fixated on the Departure Corridor AOI (C). On the other hand, the visual scan path contained multiple commonly used patterns between the BRITE radar (L), the ASDE radar (A), and the flight strips (S) AOIs, such as SAL, SA, and AS, that can be observed in other visual scan paths (e.g., S2, S8, and S5). In addition, it also contained the pattern TS between the touchdown AOI (T) and the flight strips (S) AOI present in S3, S5, and S8.

**Figure 15:**
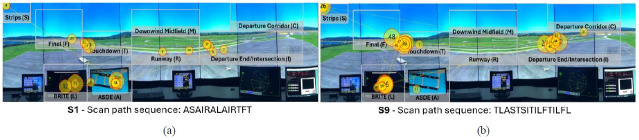
Visualization of the two visual scan paths (a-b) classified as having low string edit similarity but high Jaccard coefficient similarities overlaid the AOIs in the airport environment, alongside their scan path sequence. Note: The yellow circles denote eye fixations, and the inscribed numbers the order the said eye fixations took place. The yellow lines represent the saccadic movement of participants between eye fixations.

Figure 15 showcases the two visual scan paths classified as having low string edit similarity but high Jaccard coefficient similarity. Although the visual scan paths contain the Intersection AOI (I) and the Touchdown AOI (T), as well as both radar AOIs (L and A) and the flight strips AOI (s), they were inspected in less common patterns. For example, S1 was the only visual scan path to use the pattern AIR, which was applied twice. S9 was the only participant to apply the pattern ITI when inspecting the runways.

**Figure 16:**
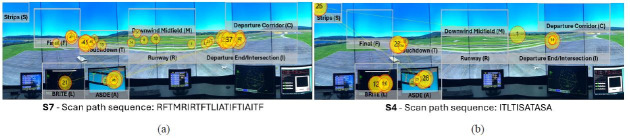
Visualization of the two visual scan paths (a-b) classified as having low similarity across both similarity metrics overlaid the AOIs in the airport environment, alongside their scan path sequence. Note: The yellow circles denote eye fixations, and the inscribed numbers the order the said eye fixations took place. The yellow lines represent the saccadic movement of participants between eye fixations.

Figure 16 visualizes the two visual scan paths classified as having low similarity across both string edit similarity and Jaccard coefficient similarity metrics. Both visual scan paths do not contain commonly inspected AOIs, such as the flight strips AOI (S) in the case of S7, as well as the Runway AOI (R) and the Final AOI (F) in the case of S4. In addition, S7 was the visual scan path to fixate on the Downwind Midfield AOI (M). Furthermore, neither visual scan path contains common AOI patterns or variations between the ASDE radar (A), the flight strips (S), and the BRITE radar (L), such as SAL or LSA, observed in other visual scan paths. On the other hand, they apply uncommon patterns, such as IAI, only applied by S4, as well as RIR, which can only be observed in S7.

## Discussion

We were able to identify similar visual scan paths carried out by expert tower controllers while issuing clear to take off clearances using our proposed framework. More specifically, our proposed approach classified the visual scan paths of tower controllers based upon their average Jaccard coefficient similarity, which accounts for the areas inspected by the controllers, as well as by their average string edit similarity, which considers the order in which the controllers inspected the areas. The contribution of the proposed framework might help researchers and practitioners interested in identifying similar visual scanning behaviors, particularly in cases where multiple variations of a similar behavior may exist.

### A. Identifying similar visual scanning strategies when multiple variations are possible

The use of two complimentary similarity metrics allowed us to identify participants with similar visual scan paths that would otherwise be classified different by using only one metric. For example, had we only applied the string edit similarity, we might have interpreted that S9 had a different visual scanning behavior even though they have a higher-than-average Jaccard coefficient similarity. In other words, throughout the visual scan path, the controller inspected many of the same AOIs as other the other participants but applied a different order. In addition, if we had only used the Jaccard coefficient similarity, we might have erroneously considered S6 to have a different visual scanning behavior even though they share many similar patterns between AOIs with other participants, as shown by their higher-than-average string edit similarity. Therefore, by combining multiple similarity metrics that complement each other we were able to correctly identify similar visual scan paths while accounting for potential variations that might exist between them.

In addition, average Jaccard coefficient similarity and string edit similarity values calculated between the expert tower controllers were higher than those reported in other tasks and environments. More specifically, we calculated an average Jaccard coefficient similarity of 0.742 and a string edit similarity of 0.322. On the other hand, in a prior study in which participants observed images, such as paintings, reported a string edit similarity of 0.28, as well as an average similarity value of 0.54 based upon the common AOIs, observed between participants on the same images (33). Another study where participants were instructed to visually fixate on letters or numbers in a pre-determined order reported an average string edit similarity of 0.23 and an average similarity value of 0.47 based upon the common AOIs that were observed by participants (7). Furthermore, one potential reason behind these differences was briefly discussed by Duchowski et al (7) is that the task participants are assigned with completing might impact how they carry out their eye movements, and as a result, the similarity values that can be calculated. Nonetheless, we also believe that the expertise of tower controllers in our experiment might have contributed to larger similarity values. The expert controllers, due to their years of experience and familiarity with the tower cab environment, know what information they need prior to issuing an aircraft to take off, as well as where that information is located in the environment. Note that these prior studies did not specifically use the Jaccard coefficient similarity, however, they did calculate the similarity of visual scan paths based upon the AOIs they share, and thus, the resulting values should be closely related.

Overall, researchers might need to account for and consider how Jaccard coefficient similarity and string edit similarity values might be impacted by the task, environment, as well as the participants’ expertise when comparing similar visual scanning patterns in order to identify similar ones. Furthermore, using multiple similarity metrics simultaneously might be particularly useful in tasks where participants can have multiple variations of similar visual scanning behaviors.

### B. Similarities in the visual scan paths of tower controllers when issuing clear to take off clearances

Most of the visual scan paths of tower controllers (6 out of 9 visual scan paths) had an above average Jaccard coefficient similarity, which aligns with prior research discussing that some AOIs in the tower cab environment have a high probability of being observed while issuing clearances. Manske & Schier (25) found that AOIs in the tower cab environment such as the flight strips, radar displays, and runways, have a higher probability of being observed by tower controllers while issuing a clear to take off than other AOIs. In our study, all visual scan paths contained both radar AOIs (A and L), runway AOIs (T, R, and I), some of the commonly inspected AOIs in the work of Manske & Schier (25). Nonetheless, these results might not be unexpected, as controllers have to inspect the runway to ensure that that there are no hazards on the runway that might lead to, for example, runway incursions (10).

Controllers in our study gathered the information required to issue a clear to take off from multiple AOIs that provided the same or similar information. Prior studies have described that, depending on the situation, controllers might look directly at the runway or the ground radar to ensure there are no potential hazards on the runway while issuing a clear to land clearance (36). In other words, two areas of the tower cab environment that provide the same information (i.e., the runway is clear) to the controller. A similar behavior might be applied by controllers when issuing a clear to take off clearance. For example, one visual scan path did not contain the flight strips AOI (S7), while another visual scan path did not contain the runway intersection AOI (S6), two of the most commonly observed AOIs by the controllers. However, these controllers were able to gather the information required to issue the clearance by observing other AOIs, such as the ASDE radar (A), which contains the callsign of the aircraft that was ready for departure, rather than looking at the flight strips, as well as to determine that there were no hazards present on the runway for the aircraft. Furthermore, participants inspected AOIs that contained the same information multiple times, such as the runway hotspot AOIs (T and I) as well as the ground radar AOI (A).

In addition, over half of the controllers (5 out of 9 controllers) applied similar visual search patterns when gathering information from the tower environment to issue a clear to take off clearance. As a result, these controllers’ visual scan paths had an above average string edit similarity. One of the common patterns applied by these controllers was to inspect the radar AOIs (L and A) one after the other (i.e., AL or LA), after which they scanned scan the hotspot AOIs (T and I) back and forth (i.e., TIT). These can be seen particularly in the case of S5 and S3, where S5 contains the sequence “LATITS” while S3 contains the sequence “LALFTITS”, which have a string edit similarity of 0.75 between each other. The other controllers had similar variations of these patterns, with S8 applying “LAITS” and S2 using the pattern “ALFTIT”.

Inspecting radar AOIs first, followed by runway hotspot AOIs, or the other way around, might serve as a way for controllers to build “the picture” (i.e., their situational awareness) (30) of the airport environment, and immediately validating the picture with new sources of information. Thus, the order the expert inspects the AOIs might not be as important as long as the required information is gathered in a timely manner. As a result, a visual scan path such as S4 indicates that the controller first gathered information on the hotspot AOIs, after which they scanned the flight strips and the ground radar. In other words, a different order from the very similar pattern applied by S5 and S3. Nonetheless, regardless of the pattern applied, if any mismatch between their mental picture and the information they are using to validate it were to occur, the controller has an opportunity to quickly instruct the pilots to disregard the clearance without affecting the movement or safety of aircraft.

### C. Limitations & future research

The proposed framework focused on comparing visual scan path sequences by considering the information inspected by the participants (Jaccard coefficient similarity) as well as the order said information was inspected in (string edit similarity). However, other characteristics of visual scan paths that should be further investigated, such as how much time was spent inspecting each AOI, or the overall geometric shape used to inspect the AOIs, might provide additional information that could be used to better determine whether two visual scan paths are similar. Researchers have proposed algorithms such as FuncSim (12), MultiMatch ( 15; 13), or ScanMatch (Cristino et al., 2010), which are capable of considering the duration of eye fixations or the shape the visual scan path when calculating the similarity between two scan path sequences. Thus, the use of these metrics might complement our classification framework by providing additional information to identify controllers with similar scanning behaviors. For example, ScanMatch considers duration when determining the similarity between visual scan path squences by repeating AOIs in the scan path sequence based upon how long they were fixated on (Cristino et al., 2010). On the other hand, MultiMatch creates vector representations of visual scan paths that allows the algorithm to compare the geometric shape of these vectors (15).

In addition, the ability of the proposed framework to distinguish between visual scan paths collected from multiple different tasks and/or environments simultaneously should be investigated. In the present work, the proposed framework was applied to the visual scan paths of tower controllers with a similar level of expertise during one specific task (issuing a clear to take off clearance) with the same scenario characteristics (e.g. the number of aircraft and their locations in the environment). Given that factors such as the task to be completed might influence the eye movements of participants (6; 2), considering visual scan paths from multiple tasks simultaneously might limit the ability of the proposed framework to identify similar visual scan paths (i.e. visual scan paths with common patterns in one task but not in another task). Future research should investigate the sensitivity of the proposed framework when different tasks or other factors that might influence eye movements are used simultaneously.

Furthermore, the proposed framework calculated the average string edit and Jaccard coefficient similarity values between participants to classify visual scan paths into one of the four categories shown in Figure 2. However, there might be other approaches that could be used to determine threshold values that could be to classify visual scan path as similar or not that are left as future research. For example, it might be possible to calculate an expected string edit and Jaccard coefficient similarity values of visual scan path sequences given a particular task (e.g. the number of AOIs present in the environment) and participant expertise (e.g. the probability of inspecting AOIs). Visual scan paths above this cut-off value might be more similar than we expected them to be, while those scan paths below the cut-off are less similar than expected. Visual scan paths above this cut-off value might be more similar than we expected them to be, while those scan paths below the cut-off are less similar than expected. Both cases might be interesting for researchers to explore further.

Lastly, the present work focused on the visual scanning strategies of a small sample of experts issuing a clear to take off clearance in one simulated airport environment. Future research is needed to evaluate the generalizability of these initial insights to the broader population of tower controllers. In addition, in order to safely manage traffic, controllers issue several different types of clearances, such as clear to land, line up and wait, among others, could also be investigated. Furthermore, what the visual scanning strategies of experts are after issuing a clearance, such as to ensure compliance by the aircraft that received the clearance should be investigated.

### Acknowledgements

This research was completed with funding from the Federal Aviation Administration (FAA) NextGen Human Factors Division (ANG-C1) in support of technical sponsors in the FAA Air Traffic Organization (ATO). The views expressed herein are those of the authors and do not reflect the view of the United States (U.S.) Department of Transportation (DOT), FAA, or The University of Oklahoma. In addition, we would also like to thank all anonymous reviewers for sharing their thoughts and providing helpful feedback.

### Ethics and Conflict of Interest

The author(s) declare(s) that the contents of the article are in agreement with the ethics described in http://biblio.unibe.ch/portale/elibrary/BOP/jemr/ethics.html and that there is no conflict of interest regarding the publication of this paper.
